# Lymphomatoid Papulosis With DUSP22 Rearrangement in a Patient With a Historical Diagnosis of Primary Cutaneous Anaplastic Large Cell Lymphoma

**DOI:** 10.7759/cureus.66022

**Published:** 2024-08-02

**Authors:** FNU Monika, Shuai Li, Emily Ambler, David Cantu, Andrew Siref

**Affiliations:** 1 Department of Pathology, Creighton University School of Medicine, Omaha, USA; 2 Department of Pathology - Hematopathology, Creighton University School of Medicine, Omaha, USA; 3 Department of Pathology - Hematopathology and Dermatopathology, Creighton University School of Medicine, Omaha, USA

**Keywords:** alcl, cd30, t-cell receptor, epidermotropism, dusp22 rearrangement, lymphoproliferative disorder

## Abstract

Lymphomatoid papulosis (LyP) with *DUSP22* rearrangement is an uncommon subtype of lymphomatoid papulosis featured histologically by two distinct patterns of epidermotropic cells, weakly CD30+ small- to medium-sized T-cells and a dermal infiltrate of strongly CD30+ medium- to large-sized T-cells. *DUSP22* rearrangement is detected more frequently in anaplastic large cell lymphoma (ALCL) than in LyP. Primary cutaneous anaplastic large cell lymphoma (pcALCL) cases can also show a similar biphasic CD30 staining pattern. LyP with *DUSP22* rearrangement has a more indolent clinical course than pcALCL and is more likely to regress without treatment. Herein, we report a unique case of LyP with *DUSP22* rearrangement diagnosed in an 81-year-old female with a historical diagnosis of pcALCL, made 20 years prior.

## Introduction

Lymphomatoid papulosis (LyP) belongs to the spectrum of primary cutaneous CD30-positive T-cell lymphoproliferative disorders (LPDs). The CD30+ LPDs include primary cutaneous anaplastic large cell lymphoma (pcALCL), LyP, and borderline cases. A designation of ‘borderline’ is reserved for cases in which the clinicopathologic features are not definitive [1]. Clinically, LyP presents with an indolent course of self-remitting localized, clustered, or more generalized small papules or nodules which can occur anywhere, but most frequently present on the trunk or extremities. Lesions can ulcerate and scar as they resolve. LyP has diverse and variable histomorphologic features that may mimic other CD30+ LPDs and lymphomas, leading to misdiagnosis or misclassification. Based on the histologic and immunophenotypic features, six types of LyP are officially recognized by the WHO classification: types A-E and LyP with *DUSP22* rearrangement [2].

First recognized in 2013 by Karai et al., LyP with *DUSP22* rearrangement is a rare variant of LyP, which was included in the updated WHO‐European Organisation for Research and Treatment of Cancer (EORTC) classification for primary cutaneous lymphomas in 2018 [2,3]. In addition to its chromosomal rearrangement involving the *DUSP22* locus on 6p25.3, a characteristic biphasic pattern of epidermotropic cells, weakly CD30+ small- to medium-sized T-cells and a dermal infiltrate of strongly CD30+ medium- to large-sized T-cells have also been described [4-6]. Large, atypical dermal lymphocytes may mimic pcALCL and large cell transformation in mycosis fungoides (MF). Correlating the histologic findings with the clinical features and course is also helpful in establishing the appropriate diagnosis [2,4].

So far, less than 20 cases of LyP with *DUSP22* rearrangement have been reported in the literature [3,4,7-9]. Herein, we report an additional case, identified in a patient previously diagnosed with pcALCL 20 years prior.

## Case presentation

**Figure 1:**
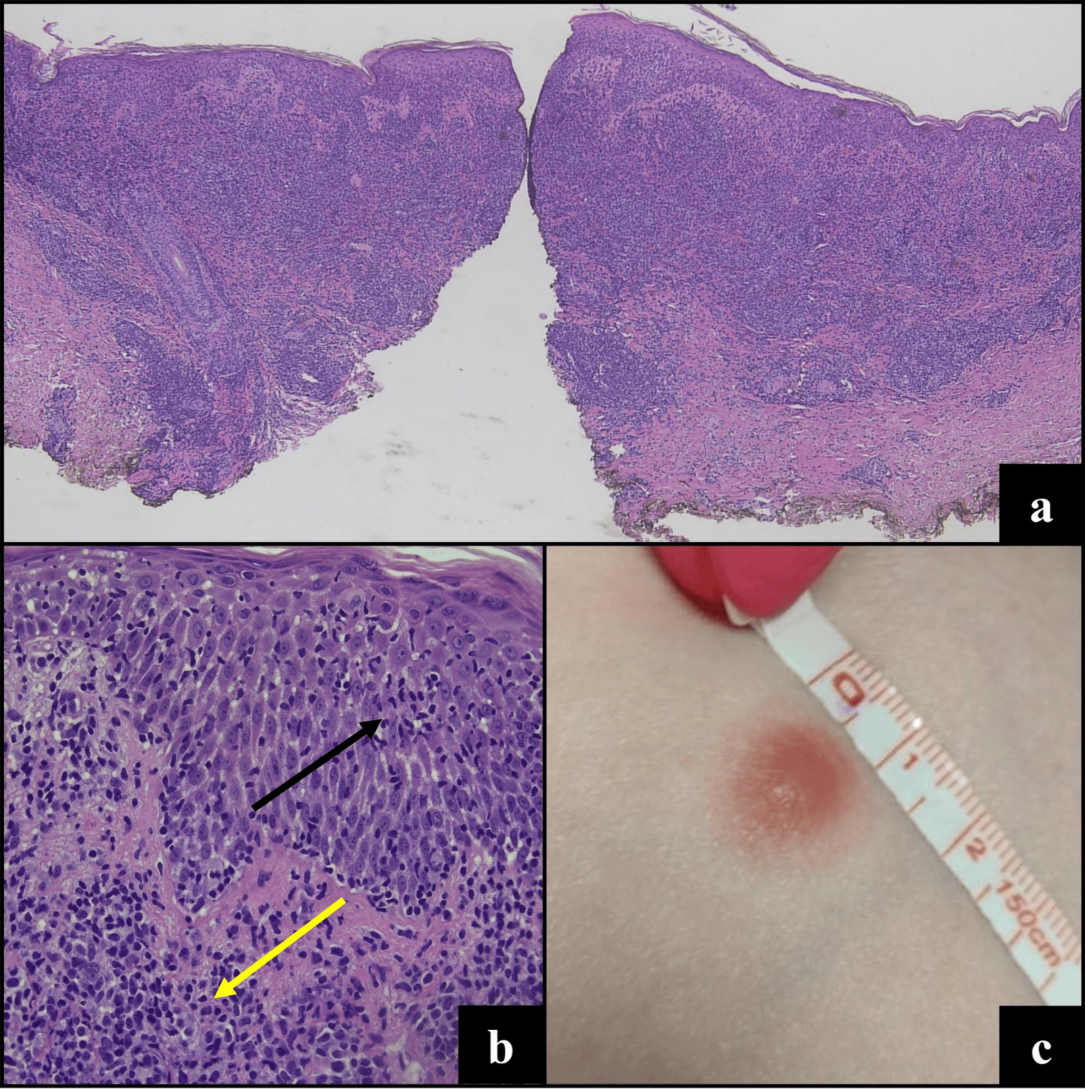
Clinical and histopathologic features of lymphomatoid papulosis (LyP) with DUSP22 rearrangement. Biopsy (H&E) specimen shows a wedge-shaped infiltrate of atypical lymphocytes with a biphasic growth pattern (a, 4x), of epidermotropic small atypical lymphocytes with irregular nuclear contour (black arrow) and the dermal component of medium- to large-sized atypical lymphocytes (yellow arrow) (b, 20x). Clinical image (c) shows a single erythematous papule on the abdomen.

An 81-year-old female presented with an erythematous papule on the abdomen, which had been noticed four days prior. On exam, the papule measured 10 mm in greatest dimension (Figure 1c). No other papular or erythematous lesions were present. A punch biopsy of the lesion demonstrated a dense, wedge-shaped atypical lymphoid infiltrate with epidermotropism (Figure 1a). The infiltrate in the dermis was composed of medium-large lymphoid cells with irregular and hyperchromatic nuclei, rare conspicuous nucleoli, and abundant cytoplasm. The epidermotropic cells were mostly small lymphocytes with less cytoplasm (Figure 1b).

**Figure 2:**
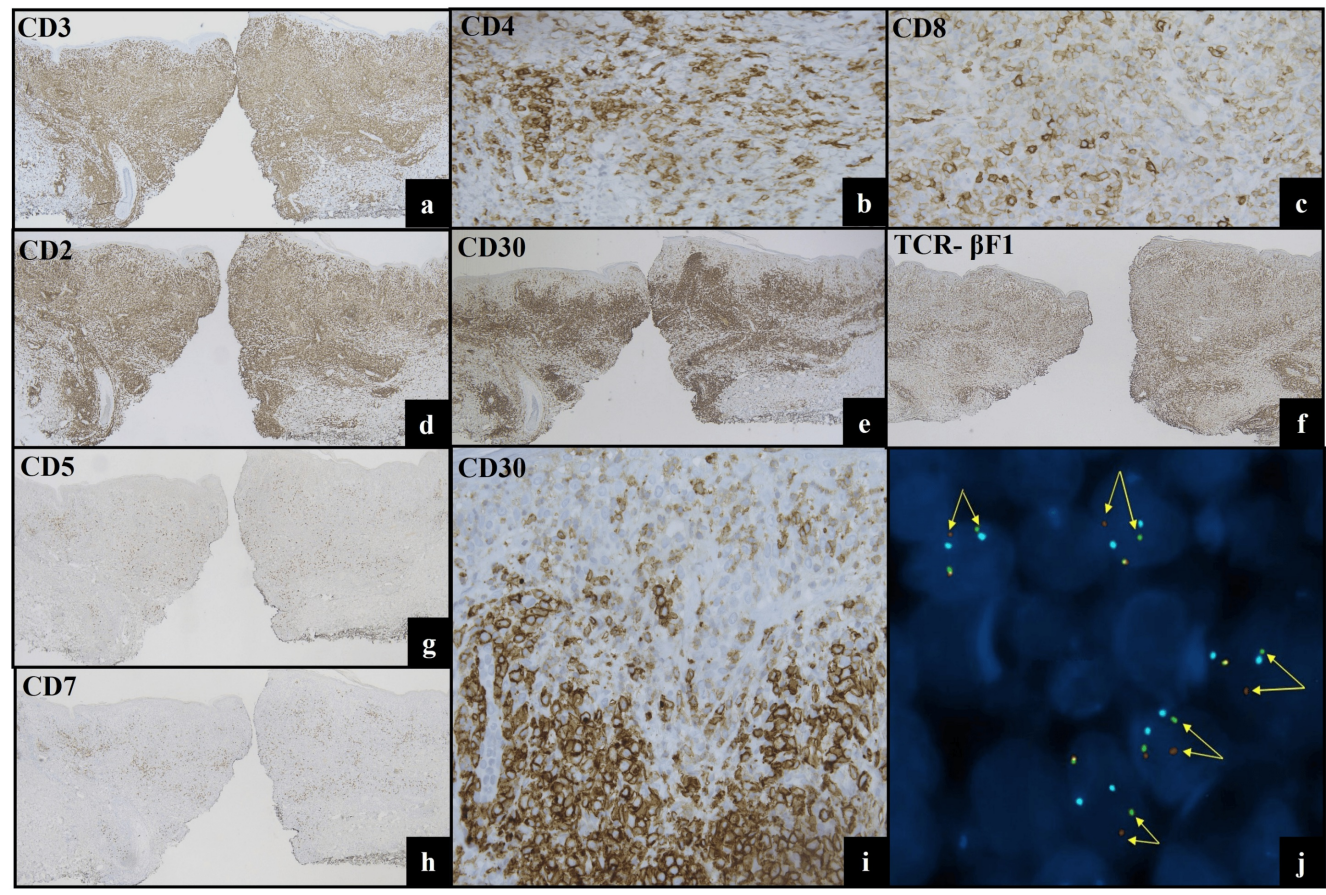
Immunophenotypic and molecular features of lymphomatoid papulosis (LyP) with DUSP22 rearrangement. Immunohistochemistry (IHC) (a-i) shows lesional cells are positive for CD3 (a, 4x), CD4 (b, subset, 40x), CD8 (c, 40x), CD2 (d, 4x), and TCR- βF1 (f, 4x) and negative for CD5 (g, 4x) and CD7 (h, 4x). CD30 is diffusely positive in both epidermal and dermal components with a “biphasic” staining pattern (e (4x), i (40x)). Fluorescence in situ hybridization (FISH) using the *DUSP22/IRF4* dual color break-apart probe reveals aberrant separation of centromeric (green) and telomeric (red) fluorescent signals with CEP 6 (6p11.1-q11.2) (aqua), confirming the presence of *DUSP22–IRF4* rearrangement (j).

Mitotic figures were easily identifiable in the dermal infiltrate. Definitive “hallmark” cells of anaplastic large cell lymphoma (ALCL) with reniform nuclei were not identified. By immunohistochemistry, the large and small cells were positive for CD3, CD2, MUM1, CD8 (weak), CD4 (in a subset), and TCR-βF1, with loss of CD5 and CD7. CD30 expression was strong in the large dermal lymphocytes but weak in the small epidermotropic lymphocytes (Figure 2a-2i). TIA-1 was expressed in a minority of cells in the infiltrate; there was no expression of granzyme B. TCR-CGM1 and ALK were also negative. Based on the morphologic features and immunophenotype, fluorescence in situ hybridization (FISH) testing was performed, which detected a rearrangement of *IRF4/DUSP22* (6p25.3) locus in 71% of interphase cells of biopsy specimen (Figure 2j). T-cell receptor gamma gene rearrangement assay identified a clonal T-cell population, which was identical to the clone demonstrated in the prior pcALCL.

**Figure 3:**
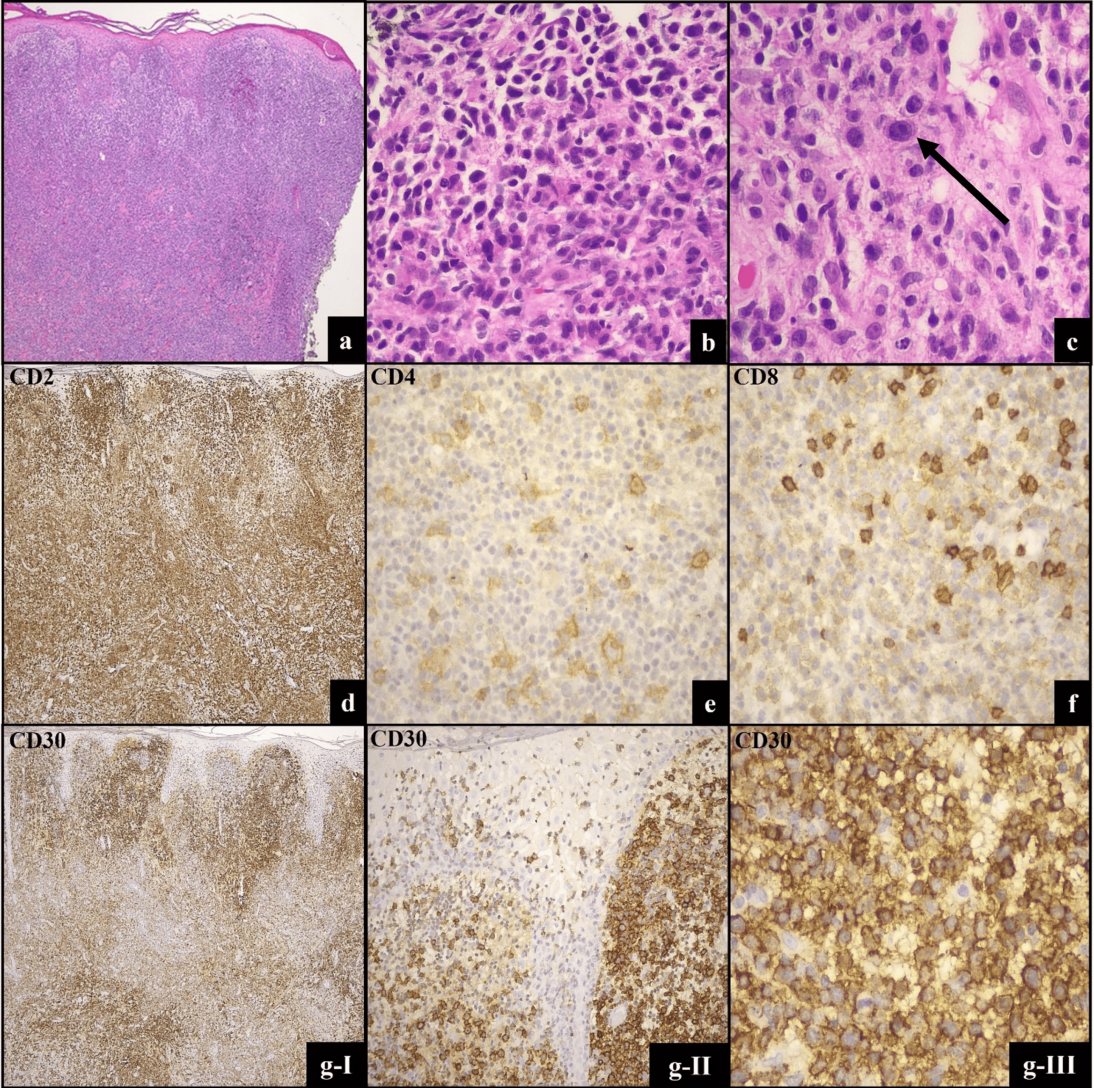
Pathologic features of primary cutaneous anaplastic large cell lymphoma (pcALCL). Dermal infiltrate of pleomorphic cells (H&E; a,4x, b,20x & c, 40x). By immunohistochemistry (IHC), tumor cells are immunopositive for CD2 (d, 4x) and CD30 (gI-III, 4x, 10x, and 40x, respectively). CD4 highlights large cells (e, 40x). CD8 is weakly positive in most cells, and strongly positive in background cells (f, 40x).

The archival pcALCL tissue block and initially generated slides (H&E, CD3, CD20) were previously discarded per hospital policy, unfortunately precluding *DUSP22* FISH testing. Some archived slides were maintained by another institution that had originally established the diagnosis of pcALCL in consultation. These archival slides were generously sent for review. The histologic sections showed a dense dermal infiltrate with some large pleomorphic cells with hyperchromatic and irregular nuclei. Epidermotropism of medium-sized cells was also present (Figure 3a-3c). The infiltrate showed a similar immunophenotype to the current lymphoid proliferation, with global loss of CD5 and CD7. The original pathology report indicated the cells were CD3 and CD2 positive (Figure 3d). However, this biopsy showed a more prominent population of CD4+ larger, pleomorphic cells (Figure 3e); these cells were not noted in the current biopsy. CD8 was weakly positive in most cells, and strongly positive in background cells (Figure 3f). CD30 expression did not readily show differences in staining quality between the dermal and epidermal lymphocytes (Figure 3gI-3gIII).

At the time of this prior biopsy, the patient endorsed other lesions, which would apparently regress. While this implicates an underlying LyP, no biopsies had been taken from other lesions.

The overall clinicopathologic findings in our current biopsy were in keeping with LyP with *DUSP22* rearrangement. Recurrent pcALCL must also be considered, however the clinical presentation of the current lesion was in contrast to the pcALCL that was reportedly ulcerated, larger (2-3 cm), and did not regress. The pcALCL was treated with external beam radiation therapy and no other clinically apparent lesions were documented during this time period; the patient also underwent yearly surveillance CT scans for 18 years without evidence of disease. The current lesion completely resolved following the biopsy, and no other manifestations or recurrence of LPD were present at the time of her most recent follow-up (12 months).

## Discussion

*DUSP22* rearrangement has been detected more frequently in ALCL than in LyP, with 20% to 30% more frequently in both pcALCL and systemic ALCL [10]. pcALCL can also show the biphasic CD30 staining pattern [4-6]. Although the biphasic pattern of CD30 staining was not seen in the pcALCL, this does not preclude the possibility of *DUSP22* rearrangement, as not all cases have clearly shown this CD30 staining pattern [4-6]. A significant limitation to any direct comparison of CD30 expression in these cases is that the stains were performed at different institutions, essentially 20 years apart.

The distinction between *DUSP22* rearranged LyP and pcALCL cannot be made solely on histopathologic evaluation alone and requires clinicopathologic correlation [4]. Our case was particularly challenging, given the historical context of a pcALCL diagnosis, established prior to the recognition of *DUSP22* rearrangement as a recurrent abnormality. Moreover, the historical diagnosis of pcALCL could not be definitively confirmed in our review. Although some larger cells were present, no obvious “hallmark” or other cells with similar cytomorphology were appreciated. This lesion apparently had worrisome clinical features, namely size and ulceration. There was therefore some uncertainty about the historical diagnosis of pcALCL in this patient.

While most patients with LyP have a generally indolent course, a number of patients (ranging from 15-50%) can develop an associated lymphoma before, after, or even during the course of the disease [1]. Although our patient may have had stigmata of LyP around the time of her pcALCL diagnosis, no other lesions were clinically documented during an apparent 20-year hiatus. It is seemingly unlikely that no such lesions arose during this timeframe; however, smaller lesions with a propensity for spontaneous resolution may not have been brought to clinical attention.

In T-cell lymphomas, monoallelic 6p25.3 alterations cause down-regulation of the dual specificity phosphatase 22 (DUSP22) protein [11]. *DUSP22* has been shown to inhibit T-cell antigen receptor signaling in reactive T-cells through inactivation of MAPK and ERK2 [12]. Otherwise, little is known about the role of *DUSP22* in physiologic and pathologic pathways; evidence suggests it may function as a tumor suppressor in neoplasia [11,13].

In LyP, there is a particular propensity for the development of clonally related MF or pcALCL [1,14]. *DUSP22* rearrangement may also occur in a subset of MF with large cell transformation, which usually shows significant CD30 expression [15,16]. The distinction between these cutaneous T-cell lymphoproliferations may not always be so clear-cut and can at times show significant overlapping clinical and histopathologic features. Recent cases of various *DUSP22* rearranged T-cell lymphomas with features spanning a spectrum from LyP to MF to pcALCL have been described [17-19]. Another recently reported case demonstrated a gamma-delta T-cell immunophenotype [20]. These reports underscore the varied spectrum of clonal T-cell lymphoproliferations that harbor the *DUSP22* rearrangement.

## Conclusions

LyP is a complex cutaneous T-cell lymphoproliferative disorder that can develop associated lymphomas, as demonstrated in our case. Cases of LyP and pcALCL with *DUSP22 *rearrangement can show a biphasic CD30 staining pattern, a useful clue to the diagnosis. LyP with *DUSP22 *rearrangement is a diagnosis that requires clinicopathologic correlation and cytogenetic testing for confirmation.

Increased recognition of, and testing for, this LyP subtype will allow for more in-depth analysis. Further study is necessary to elucidate the role of *DUSP22 *rearrangement in T-cell lymphomagenesis and will hopefully provide a unified framework for the varied clinical and histopathologic manifestations of *DUSP22*-rearranged T-cell lymphoproliferations.
